# The Nematodes *Thelazia gulosa* Railiet and Henry, 1910 and *Thelazia skrjabini* Erschov, 1928 as a Cause of Blindness in European Bison (*Bison bonasus*) in Poland

**DOI:** 10.1007/s11686-020-00243-w

**Published:** 2020-07-01

**Authors:** Aleksander W. Demiaszkiewicz, Bożena Moskwa, Aneta Gralak, Zdzisław Laskowski, Anna W. Myczka, Marta Kołodziej-Sobocińska, Stanisław Kaczor, Elwira Plis-Kuprianowicz, Michał Krzysiak, Katarzyna Filip-Hutsch

**Affiliations:** 1 Witold Stefański Institute of Parasitology, Polish Academy of Sciences, Warsaw, Poland; 2 Mammal Research Institute, Polish Academy of Sciences, Białowieża, Poland; 3 District Veterinary Inspectorate, Sanok, Poland; 4 Białowieża National Park, Białowieża, Poland; 5 Faculty of Civil Engineering and Environmental Sciences, Institute of Forest Sciences, Białystok University of Technology, Bialystok, Poland

**Keywords:** *Bison bonasus*, Morphometric analysis, Parasitic infections, *Thelazia* spp.

## Abstract

**Purpose:**

The nematodes of the genus *Thelazia* are the cause of eye diseases of wild and domestic ruminants throughout the world. The aim of the study was to describe clinical cases of thelasiosis in European bison (*Bison bonasus*) in Poland, and provide morphometrical features of *Thelazia gulosa* Railiet and Henry, 1910 and *Thelazia skrjabini* Erschov, 1928 regarded as potentially useful for species differentiation

**Methods:**

The conjunctival sacs, tear ducts, the surface of the cornea and nicitating membrane collected from bison were rinsed with saline solution. Any nematodes isolated from the sediment were subjected to morphometric analysis.

**Results:**

Thirteen of the 16 examined European bison were infected with *Thelazia* nematodes*,* belonging to the species *T. gulosa* and *T. skrjabini.* The intensity of infection ranged from one to six (mean intensity 5), and four to 29 (mean intensity 14) nematodes *T. skrjabini* and *T. gulosa* respectively. Congestion of conjunctival sac, keratitis and corneal opacity, corneal ulceration and perforation as well as purulent eyeball inflammation were observed in infected animals.

**Conclusions:**

*Thelazia gulosa* and *T. skrjabini* can be identified by morphometrical features. As thelasiosis might be a serious threat for protected population of European bison, further studies are needed of the epidemiology and pathology of this emerging parasitosis in Poland.

## Introduction

The European bison (*Bison bonasus*) is the largest mammalian herbivore in Europe. Although it became extinct in the wild at the beginning of XX century, the species was successfully restored from captive animals and reintroduced to a number of countries in central and eastern Europe [1]. Currently, there are about 7500 European bison worldwide and over 24% of their population inhabit Poland, which remains the center of European bison breeding [2, 3]. However, the European bison remains an endangered and rare species, demanding regular health monitoring. The biggest threat for the population of European bison is presented by infectious diseases, including parasitoses [4–6]. Parasitological studies of European bison in Poland have been performed since the 1950s [7, 8]; however, climate change, animal migrations and the growth of the European bison population constantly provoke the spread of new emerging parasitoses. Recently, an increasing number of clinical cases of thelasiosis in European bison has been observed in Poland (Stanisław Kaczor, pers. commun.).

Nematodes of the genus *Thelazia* (Spirurida: Thelaziidae) are agents of eye diseases of domestic and wild ruminants in Europe, Asia, Africa, North America and Australia. The genus *Thelazia* is represented by five species in Europe: *Thelazia gulosa* Railiet and Henry, 1910, *T. rhodesi* Desmarest, 1827, *T. skrjabini* Erschov, 1928, *T. lacrymalis* Gurlt, 1831 and *T. callipaeda* Railliet and Henry, 1910. The nematodes infect cattle (*Bos taurus*), zebu (*Bos indicus*), European and American bison (*Bison bison*) and buffalo (*Syncerus caffer*), where they localize in the conjunctival sac and tear ducts, as well as beneath the nictitating membrane and on the cornea. *T. rhodesi* was also isolated from the eyeball of a horse (*Equus caballus*) [9] and *T. callipeda* from wild and domestic carnivores [10]. In addition, *T. gulosa* infection was also confirmed in a human in the United States of America [11, 12].

Flies from the genus *Musca*, including *M. amica*, *M. autumnalis*, *M. larvipara*, *M. osiris*, *M. vitripennis* and *M. hervei* are considered as intermediate hosts of *Thelazia* nematodes [9, 13, 14]. Mature female nematodes produce numerous larvae in the conjunctival sac of the definitive host. The larvae enter flies, the intermediate host, as they are licking the secretions from the eye of the host. In the fly, the larvae develop into their invasive stage by moulting twice, and then migrate to the fly suckers. From here, they are passed into the conjunctival sac of the next definitive host while the fly is licking the secretion from the eye of the ruminant [9, 15].

The pathogenic effect of nematodes of the genus *Thelazia* in cattle derives from the mechanical irritation of the conjunctiva and cornea, as well as the toxic effect of parasite metabolites. The infected animals suffer from acute conjunctivitis, tearing, swelling and increased eyelid temperature, conjunctival congestion and photophobia, as well as the production of serous and mucous exudates, which turn into purulence and cause eyelid clumping. This is followed by corneal opacity and ulceration with secondary bacterial infections, leading to purulent eye inflammation. Lesions in eyeballs are accompanied by general clinical signs, e.g. lack of appetite, indigestion and emaciation [16, 17].

The aim of the study was to describe cases of thelasiosis in European bison in Poland and to perform a morphometrical analysis of the nematodes *T. gulosa* and *T. skrjabini* to identify potentially differentiating features.

## Materials and Methods

Sixteen European bison, aged three to 20 years, were included in the study; all had died in the years 2018–2020 due to various reasons, such as weakness, skin disorders and balanopostitis. All bison came from free-living populations in the Białowieża Primeval Forest (11 animals) and the Knyszyn Forest (one animal) in north-eastern Poland as well as the Bieszczady Mountains (four animals) in southern Poland. Five of the bison in the Białowieża Primeval Forest and three in the Bieszczady Mountains were culled due to visible changes in eyeballs. The eyeballs of the examined bison, together with adjacent tissues, were delivered to the laboratory of the Witold Stefański Institute of Parasitology, Polish Academy of Sciences and subjected to parasitological examination. The conjunctival sacs, tear ducts, the surface of the cornea and the nictitating membrane were rinsed with physiological solution. The eyeballs were dissected and their structures thoroughly rinsed. The decantated sediment was examined under the stereoscopic microscope on the presence of nematodes. Any obtained parasites were isolated and stored in 70% alcohol with 5% glycerol for further identification. Morphometrical analysis of the nematodes was performed using an Olympus BX50 light microscope (Olympus, Japan) with a Cell D digital image analysis system.

## Results

Nematodes from the genus *Thelazia* were identified in 13 of the 16 examined European bison, i.e. a prevalence of 81.2%. Nematodes belonging to the species *T. gulosa* and *T. skrjabini* were found in the conjunctival sacs, tear ducts, under the nictitating membrane and on the surface of the cornea. Nine of the infected European bison came from the Białowieża Primeval Forest. Four animals were infected with *T. gulosa* and another four with *T. skrjabini*. One bison was co-infected with both *Thelazia* spp., isolated from the same eyeball: the animal was culled due to emaciation and blindness, caused by cataract and corneal ulceration. In the Knyszyn Forest, *T. gulosa* nematodes were isolated from one European bison. In the Bieszczady Mountains, three animals were infected with *T. skrjabini*. The intensity of infection ranged from one to six nematodes with mean intensity reaching five for *T. skrjabini* and four to 29 with mean intensity 14 for *T. gulosa*. The highest infection intensity was observed in a European bison from the Białowieża Primeval Forest: a 17-year-old male, culled due to skin disorders.

Five infected European bison, more specifically four from the Białowieża Primeval Forest and one from the Knyszyn Forest, did not show clinical signs of thelasiosis, and no pathological changes were found in their eyeballs during necropsy. However, numerous gross lesions were observed in the eyeballs of eight infected animals, including all three European bison examined in the Bieszczady Mountains and five from the Białowieża Primeval Forest: pathological changes included hyperemia and keratitis of the conjunctival sac, and its opacity, cataract, corneal ulceration and purulent ocular inflammation, as well as perforation of corneal ulcers.

### Morphometrical Description of *T. gulosa*

**Fig. 1:**
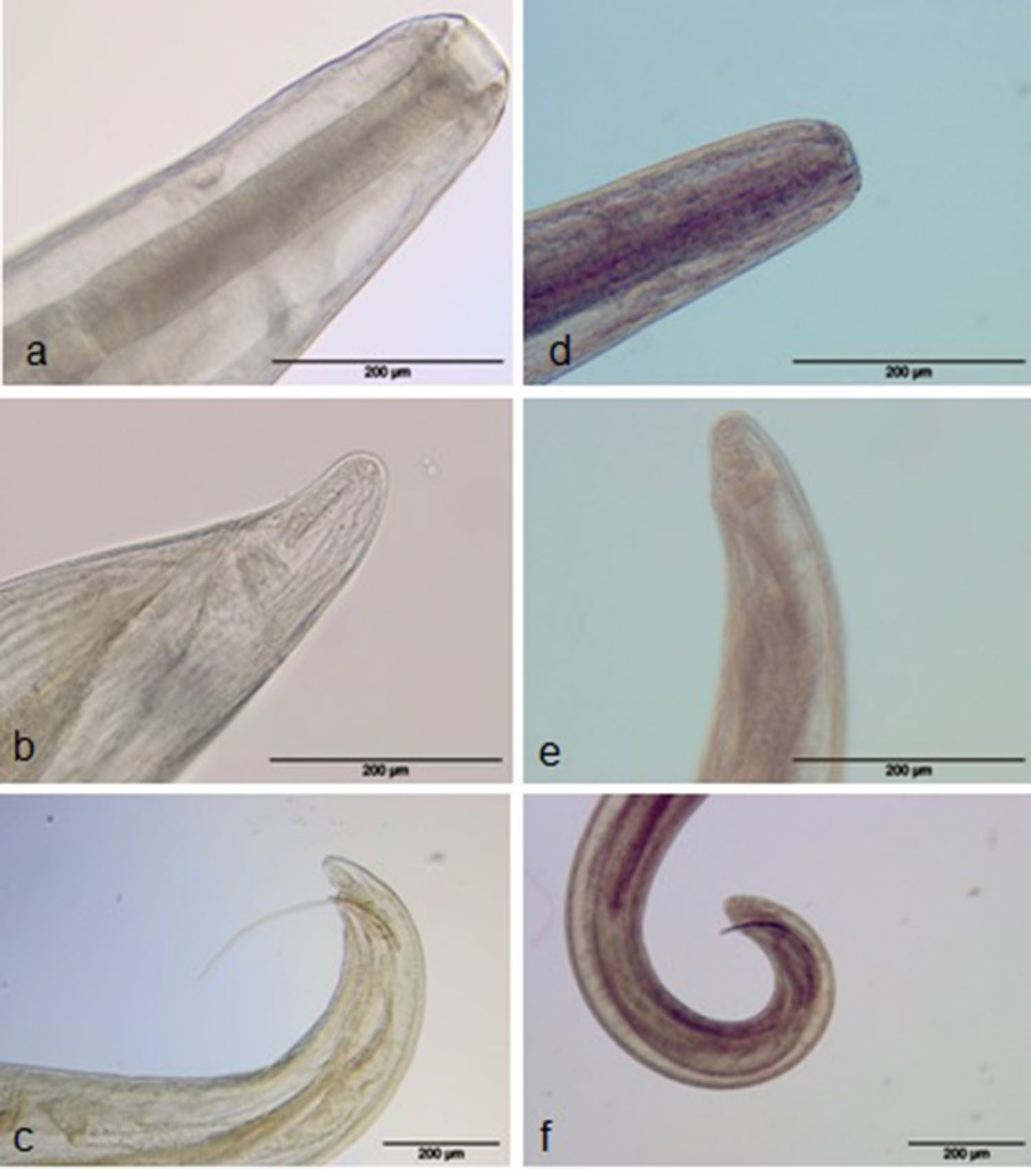
Morphometrical characteristics for the differential diagnosis of *Thelazia gulosa* (**a**–**c**) and *Thelazia skrjabini* (**d**–**f**). **a** and **d**—anterior end of the female; **b** and **e**—posterior end of the female; **c** and **f**—posterior end of the male

White-yellowish nematodes are rounded at the anterior end and pointed at the posterior one (Fig. 1). The body is covered with a cuticle with pronounced transverse striation. The lipless mouth has a well-developed buccal capsule. The opening of the mouth was surrounded by four papillae and two amphids; the mouth enters into a cylindrical pharynx, which slightly widens before entering the intestine.

Male: Body length from 6.6 to 9.8 mm, maximum width 275–489 µm, buccal capsule width 29–47 µm and buccal capsule depth 20–33 µm. The length of the cylindrical pharynx 275–380 µm**,** the width 69–83 µm. The nerve ring is located about 156–261 µm from the anterior end of the body. The body is 91–104 µm wide at the area of the cloaca. Two spicules are located at the posterior end of the body: the right spicule is shorter than the left and slightly bent, i.e. 132–163 µm long and 9–20 µm wide, while the left spicule is much longer and thinner, i.e. 745–897 µm long and 6–9 µm wide. The cloaca is located 89–104 µm from the posterior end of the body; 10 to 24 papillae are located behind the cloaca, and three pairs in front of it. The posterior end of the body is ventrically bent and pointed. The tail cuticle has two small sensilia (phasmids).

Female: The body ranges from 8.3 to 14.9 mm in length, maximum width 375–585 µm. The buccal capsule is 40–55 µm wide, and 16–41 µm deep. The cylindrical pharynx is 369–425 µm long and 73–106 µm wide. The nerve ring is located about 231–254 µm from the anterior end of the body. The genital pore is about 564–715 µm from the anterior end of the body and the anal pore 121–174 µm. from the posterior end.

### Morphometrical Description of *T. skrjabini*

The nematodes are thin and yellow with rounded, tapered ends (Fig. 1). The cuticle shows very delicate, barely visible striation. The mouth opens terminally, surrounded by two rows of papillae: the internal row consists of six small papillae located directly around the mouth, and the external row consists of four larger papillae and two amphids. The mouth leads to a small buccal capsule, maximum width 22–25 µm and maximum depth 7–8 µm.

Male. The body is 7.1–9.2 mm long and 197–211 µm wide. The pharynx is 325–386 µm long and 39–46 µm wide. The nerve ring is located about 165–192 µm from the anterior end of the body. The posterior end of the nematode is bent and equipped with two spicules; both are 8–10 µm wide, however, the right is 92–12.1 µm long and the left is 130–176 µm long. The cloaca is located 82–92 µm from the posterior end of the body. In addition, 15–30 pairs of papillae are located behind the cloaca and only three pairs in front.

Female. The body is 10.8–15.9 mm long and 235–282 µm wide. The pharynx is 312–367 µm long and 51–65 µm wide. The nerve ring is located about 171–217 µm from the anterior end of the body. The genital pore is about 474–625 µm from the anterior end of the body. The anal pore is located 91–97 µm. from the posterior end of the body. The tail is 85–93 µm long. A pair of phasmids are present at the posterior end of the nematode.

## Discussion

Little data currently exists about the occurrence of nematodes from the genus *Thelazia* in ruminants in Europe. However, infection of bovines with nematodes belonging to the species *T. gulosa* and *T. skrjabini* was commonly diagnosed between the 1940s and 1960s in many regions of Poland. The prevalence of nematodes ranged from 4.4 to 25% of examined domestic ruminants. Thelasiosis was also a cause of keratoconjunctivitis in some bovine individuals; however, the intensity of infection was low, not exceeding 15 nematodes [15–25].

Although thelasiosis had commonly been observed in bovines in Poland, only few studies have examined the infection of wild ruminants. During the years of European bison restitution, no clinical signs of parasitosis were observed in either captive or free-living animals. The first report of *T. gulosa* and *T. skrjabini* in European bison in Poland comes from 1954–1957 [7, 8]. The mentioned study showed that 16% of examined animals were infected with *T. skrjabini* and 12% with *T. gulosa*, with the infected European bison coming from the Białowieża Primeval Forest, Niepołomice Forest, Pszczyna and zoological garden in Płock.

In later years (1983–1986), *T. gulosa* nematodes were found in two out of seven examined bison in the Białowieża Primeval Forest and the intensity of infection reached only one or two nematode specimens [26]. However, no clinical signs of thelasiosis were observed in European bison examined in the 1950s and 1980s, despite them being infected with the parasite. The very first clinical case of thelasiosis was published only recently, in 2013, in European bison from the Bieszczady Mountains. Infection with nematode *T. gulosa* was a cause of serious pathological lesions, including bilateral opacity and erosions of the cornea, as well as lens damage and purulent eye inflammation, leading to the culling of the infected animal due to blindness [27].

In 2019, clinical signs of thelasiosis were observed in several bison from the Bieszczady Mountains (Stanisław Kaczor, pers. commun.) and from the Białowieża Primeval Forest (Michał Krzysiak, pers. commun.). Over 80% of European bison from three different locations, examined in the present study, were infected with *T. gulosa* and/or *T. skrjabini*. Even though the infection was accompanied by distinct, visible clinical signs in eight animals, another five infected individuals did not demonstrate any signs of the disease. Hence, the problem of thelasiosis is probably more widespread than can be determined just by observing European bison in the wild, as accurate laboratory analysis is needed to detect subclinical cases of infected animals. Presumably, the animals with no disease are at an early stage of the disease; however, they may still be a source of infection for other individuals.

It is difficult to speculate about the reason for the increasing number of clinical cases of thelasiosis observed in European bison in Poland. The transmission of the disease in the environment occurs through flies which act as an intermediate host of *Thelazia* nematodes; such transmission enables significant promotion of parasite spread and complicates any control strategies, compared to parasites with a simple life cycle, e.g. blood-sucking nematode *Ashworthius sidemi* (Strongylida; Trichostrongylidae). In case of *A. sidemi*, the highest infection intensities have been recorded in the largest bison herds, where the winter supplementary feeding of European bison is common [4]. Therefore, it may be possible to the suppress the spread of the parasite by modifying European bison management practices. Furthermore, it cannot be ruled out that higher animal densities also favour *Thelazia* spp. transmission, as previously shown for domestic cattle [23, 24].

In addition, it might be possible that the transmission is facilitated by the migration of European bison and their utilization of fields and meadows outside the forest; the same places which are grazed by domestic cattle. Currently, around 250 European bison live permanently outside the complex of the Białowieża Primeval Forest and graze on fields together with domestic ruminants (Michał Krzysiak, pers. commun.). Unfortunately, there is no recent data about the prevalence of thelasiosis in domestic ruminants in Poland; however, it should not be excluded that infected cattle may be one of the possible sources of this parasitosis for European bison. Moreover, it cannot be ruled out that climate change may have an impact on increasing parasite transmission and infectivity [28]. Flies, intermediate hosts for *Thelazia* transmission, are probably present in greater numbers and for longer durations during the year when mean annual temperatures are higher and winters are milder.

The morphometrical description of the nematodes isolated from the European bison in the present study corresponds with those given by other authors [8, 29, 30] and indicates the presence of two *Thelazia* spp. species—*T. gulosa* and *T. skrjabini*. However, all *Thelazia* spp. species exhibit the same pathogenicity [29]. Therefore, the anathomopathological changes observed in the examined European bison are similar to those described in cattle by other authors [16, 17, 25]. Extremely intensive *Thelazia* spp. infection in bovine may lead to the formation of granulomas on the conjunctival sac, together with inflammatory infiltration [30]; however, no such lesions were observed in European bison in the present study, possibly due to the low infection intensity.

## Conclusions

To conclude, the presence of nematodes from the genus *Thelazia* in the three examined wild populations of European bison suggests that the parasite may be a serious threat for this endangered species, especially when clinical signs occur. The recent increase in observations of thelasiosis in European bison is disturbing and demands regular monitoring, not only because of the high possibility of transmission of the disease to other ruminants. Further research on the epidemiology and pathology of this emerging disease in populations of both wild and domestic ruminants in Poland and other European countries is needed.

## Data Availability

The datasets generated during and/or analysed during the current study are available from the corresponding author on reasonable request.

## References

[1] Krasińska M, Krasiński Z (2013). European bison. The nature mono-graph.

[2] Raczyński J (2018). Księga rodowodowa żubrów 2018. Białowieski Park Narodowy, Białowieża.

[3] Larska M, Krzysiak MK (2019) Infectious disease monitoring of European bison (*Bison bonasus*). In: Wildlife population monitoring, IntechOpen. 10.5772/intechopen.84290

[4] Kołodziej-Sobocińska M, Demiaszkiewicz AW, Lachowicz J, Borowik T, Kowalczyk R (2016). Influence of management and biological factors on the parasitic invasions in the wild–spread of blood-sucking nematode *Ashworthius sidemi* in European bison (*Bison bonasus*). Int J Parasitol Parasites Wildl.

[5] Krzysiak MK, Jabłoński A, Iwaniak W, Krajewska M, Kęsik-Maliszewska J, Larska M (2018). Seroprevalence and risk factors for selected respiratory and reproductivetract pathogen exposure in European bison (*Bison bonasus*) in Poland. Vet Microbiol.

[6] Krzysiak MK, Demiaszkiwicz AW, Larska M, Tomana J, Anusz K (2020). Parasitological monitoring of European bison (*Bison bonasus*) from three forests of north-eastern Poland between 2014 and 2016. J Vet Res.

[7] Dróżdż J (1958). Helmintofauna żubra, *Bison bonasus* (L.), w Polsce. Wiad Parazytol.

[8] Dróżdż J (1961). A study on helminth and helminthiases in bison, *Bison bonasus* (L.) in Poland. Acta Parasitol Pol.

[9] Anderson RC (1992). Nematode parasites of vertebrates. Their development and transmission.

[10] Čabanová V, Miterpáková M, Oravec M, Hurníková JS, Nemčíková G, Brincko Červenská M (2018). Nematode *Thelazia callipaeda* is spreading across Europe. The first survey of red foxes from Slovakia. Acta Parasitol.

[11] Bradbury RS, Breen KV, Bonura EM, Hoyt JW, Bishop HS (2018). Case report: conjunctival infestation with *Thelazia gulosa*: a novel agent of human thelaziasis in the United States. Am J Trop Med Hyg.

[12] Bradbury RS, Gustafson DT, Sapp SGH, Fox M, Almeida M, Boyce M, Iwen P, Herrera V, Ndubuisi MK, Bishop HS (2020). A second case of human conjunctival infestation with *Thelazia gulosa* and a reviev of *T. gulosa* in North America. Clin Infect Dis.

[13] O’Hara JE, Kennedy MJ (1991). Prevalence and intensity of *Thelasia* spp. (Nematoda: Thelazioidea) in a Musca autumnalis (Diptera: Muscidae) population from central Alberta. J Parasitol.

[14] Otranto D, Traversa D (2005). *Thelazia* eyeworm: an original endo- and ecto-parasitic nematode. Trends Parasitol.

[15] Stefański W (1950). Parazytologia weterynaryjna wobec Kongresu Nauki. Med Wet.

[16] Kostyra J (1960). Przebieg i leczenie telazjozy u bydła. Med Wet.

[17] Rosłan J (1965). Badania nad telazjozą bydła w Polsce. Wiad Parazytol.

[18] Donigiewicz K (1946). Inwazyjne schorzenia oczu u bydła rogatego. Med Wet.

[19] Wilczyński M (1948). Pasożytnicze zapalenie spojówek bydła. Med Wet.

[20] Patyk S, Grzywiński L (1954) Masowe zachorowanie bydła na telazjozę. Pamiętnik IV Zjazdu PTP 65–66

[21] Bielawski M (1962). O leczeniu telazjozy bydła. Med Wet.

[22] Gajewski D (1963). Przypadki telazjozy bydła na terenie rzeźni warszawskiej. Med Wet.

[23] Sobolewska M, Gajda T (1970). Uwagi o wynikach leczenia telazjozy bydła. Med Wet.

[24] Kozakiewicz B (1971). Telazjoza bydła na Żuławach. Med Wet.

[25] Dróżdż J, Demiaszkiewicz AW, Lachowicz J (1989). The helminth fauna of free-ranging European bison, *Bison bonasus* (L.). Acta Parasitol Pol.

[26] Michalski L (1976). Skuteczność terapeutyczna levamisolu i tetramisolu przy telazjozie bydła. Med Wet.

[27] Demiaszkiewicz AW, Kaczor S (2015). Przypadek telazjozy u żubra w Bieszczadach. Życie Wet.

[28] Kołodziej-Sobocińska M (2019). Factors affecting the spread of parasites in population s of wild European terrestial mammals. Mammal Res.

[29] Naem S (2007). Morphological differentiation among three *Thelazia* species (Nematoda: Thelaziidae) by scaning electron microscopy. Parasitol Res.

[30] Hassan EB, Moshaverinia A, Sheedfar F, McCovan Ch, Bazargani TT, Hosseinzadeh A, Saghari R, Ashrafihelan J, Beveridge I (2017). A report of the unusual laesions caused by *Thelazia gulosa* in cattle. Vet Parasitol Reg Stud Rep.

